# m^6^A-induced LINC00958 promotes breast cancer tumorigenesis via the miR-378a-3p/YY1 axis

**DOI:** 10.1038/s41420-020-00382-z

**Published:** 2021-02-02

**Authors:** Dongwen Rong, Qian Dong, Huajun Qu, Xinna Deng, Fei Gao, Qingxia Li, Ping Sun

**Affiliations:** 1 Department of Oncology, the First Hospital of Shanxi Medical University, Taiyuan, Shanxi Province 030012 China; 2 Department of Internal Oncology, The Fourth Hospital of Hebei Medical University, Shijiazhuang, Hebei Province 050010 China; 3 Department of Oncology, Yantai Yuhuangding Hospital, Yantai, Shandong 264000 China; 4 The Fourth Department of Oncology, Hebei General Hospital, Shijiazhuang, Hebei Province 050051 China

**Keywords:** Apoptosis, Breast cancer

## Abstract

Increasing evidence demonstrates that long noncoding RNAs (lncRNAs) play critical roles in human breast cancer (BC) tumorigenesis. However, the mechanisms by which lncRNA and N^6^-methyladenosine (m^6^A) regulate BC tumorigenesis are still unclear. In the present research, LINC00958 was markedly overexpressed in BC tissue and cells, and LINC00958 upregulation promoted the tumor progression of BC cells. Mechanistically, m^6^A methyltransferase-like 3 (METTL3) gave rise to the upregulation of LINC00958 by promoting its RNA transcript stability. Moreover, LINC00958 acted as a competitive endogenous RNA for miR-378a-3p to promote YY1. Overall, these data provide novel insight into how m^6^A-mediated LINC00958 regulates BC tumorigenesis.

## Introduction

Breast cancer (BC) is one of the malignancies with the highest mortality worldwide and is thus one of the leading causes of cancer-related death among women^1,2^. Globally, although remarkable advancements have been achieved in diagnosis and clinical therapeutics, the lethality of BC is still severe^3,4^. An increasing number of clinical tactics illustrate that accurate etiological treatments are of the utmost importance in inhibiting BC mortality; however, they are hampered by the deficiency of effective therapeutic targets^5–7^. Thus, it is notably necessary to identify the molecular mechanism of BC to generate tailored targeted therapies.

Long noncoding RNAs (lncRNAs) are defined as noncoding transcripts and thus do not encode proteins^8,9^. LncRNAs are a large group of RNA transcripts longer than 200 bp that are expressed in highly divergent eukaryotes with tissue specificity. Indeed, lncRNA expression levels are altered under specific pathological conditions^10^. With the rapid development of RNA sequencing and bioinformatics, many lncRNAs have been identified as localized and tissue-specific lncRNAs. For example, lncRNA CASC15 functions as a competing endogenous RNA of miR-153-3p and targets the 3′-UTR of KLF5 mRNA, and the transcription factor KLF5 then combines with the promoter region of CASC15 to promote its expression^11^. In triple-negative breast cancer, a higher tumor LINC00173 level indicates worse survival compared to that of patients with lower levels, and silencing LINC00173 inhibits colony formation, proliferation, and invasion^4^.

*N*^6^-methyladenosine (m^6^A) is one of the most abundant internal mRNA modification forms in eukaryotes^12^. m^6^A modification is important for posttranscriptional regulation. Recent discoveries of m^6^A methyltransferases and demethylases indicate that m^6^A is a reversible and dynamic modification process^13^. In BC, m^6^A methyltransferase-like 3 (METTL3) is upregulated and regulates apoptosis and tumor growth by targeting Bcl-2^14^. Moreover, m^6^A-RNA immunoprecipitation sequencing (MeRIP-Seq) identifies the axis of FTO-BNIP3, which regulates BC cell proliferation in vitro and metastasis in vivo^15^.

Increasing evidence has indicated that lncRNAs play more critical roles in BC biological processes, including differentiation, adipogenesis, proliferation, and metastasis. LINC00958 is a canonical lncRNA in human cancer progression. For example, LINC00958 is significantly overexpressed in cervical cancer tissues and cells and promotes cervical cancer cell metastasis and proliferation by sponging miR-625-5p/LRRC8E^16^. However, in BC tumorigenesis, there is still no evidence to illustrate the role of LINC00958 in this progression. Thus, our study focuses on the potential roles of LINC00958.

In our present study, our research revealed that lncRNA LINC00958 was significantly upregulated in BC tissue and cells. Functional assays found that LINC00958 upregulation regulated the proliferation and apoptosis of BC cells. Mechanistically, METTL3-mediated m^6^A modification promotes the upregulation of LINC00958 by promoting its RNA transcript stability. Moreover, LINC00958 acted as a competitive endogenous RNA (ceRNA) for miR-378a-3p to positively regulate YY1. Taken together, these findings provide novel insight into how m^6^A-mediated lncRNAs regulate BC tumorigenesis and provide a potential therapeutic target for BC.

## Results

### The lncRNA LINC00958 indicated an unfavorable prognosis for patients with BC

**Fig. 1:**
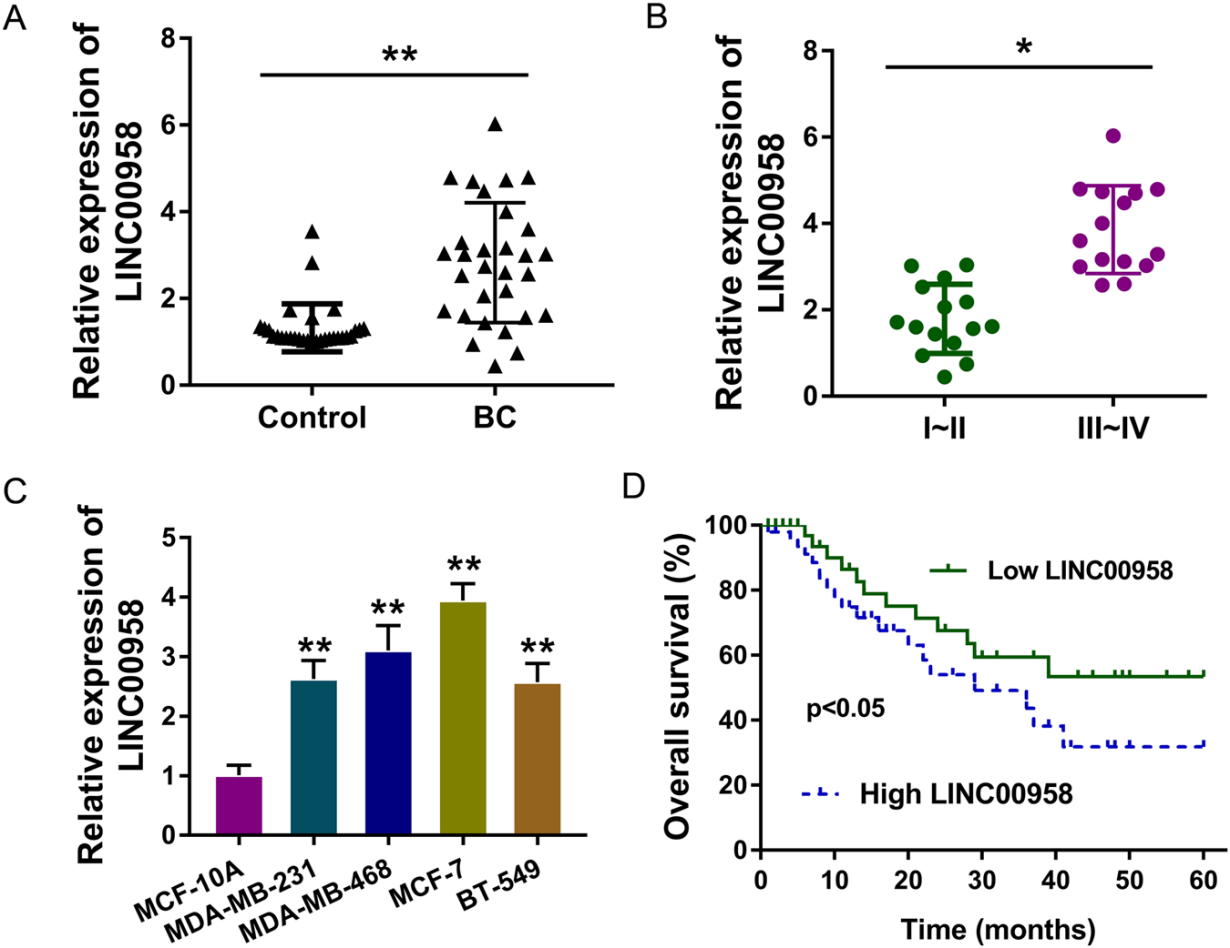
LncRNA LINC00958 indicated an unfavorable prognosis for patients with BC. **A** The expression of LINC00958 was detected using RT-PCR in the enrolled patients with BC and control subjects. **B** The BC samples were divided into two groups (I/II and III/IV) according to clinical stage. RT-PCR was performed to detect LINC00958 expression. **C** RT-PCR indicates the levels of LINC00958 in normal mammary cells (MCF-10A) and BC cell lines (MDA-MB-231, MDA-MB-468, MCF-7, and BT-549). **D** Survival rate analysis reveals the unfavorable prognosis of BC clinical patients with higher LINC00958 expression. *P* < 0.05 was considered as significant.

Among the enrolled patients with BC, the expression of LINC00958 was detected and found to be markedly upregulated (Fig. 1A). Moreover, these samples were divided into two groups according to clinical stage, and the results demonstrated that LINC00958 expression was higher in the advanced stage (III–IV grade) than in the primary stage (I–II grade) (Fig. 1B). On the basis of clinical samples, we also detected the levels of LINC00958 in BC cell lines and found that LINC00958 was markedly upregulated compared to that in normal cells (Fig. 1C). Survival rate analysis found that higher LINC00958 expression indicated an unfavorable prognosis for patients with BC (Fig. 1D). Taken together, these results showed that the lncRNA LINC00958 indicated an unfavorable prognosis for patients with BC.

### m^6^A induced the upregulation of LINC00958 in BC cells

**Fig. 2:**
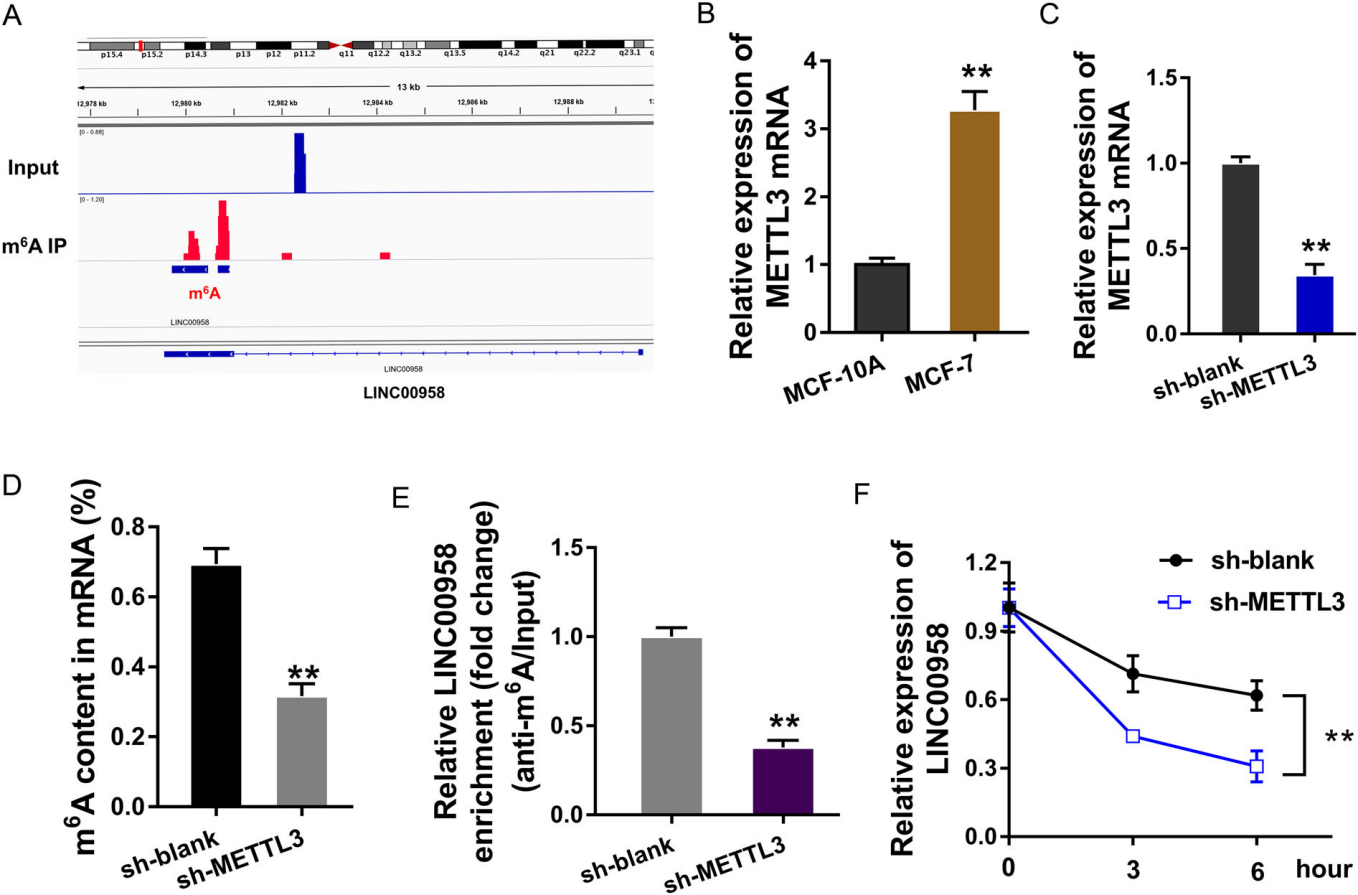
m^6^A induced the upregulation of LINC00958 in BC cells. **A** MeRIP-Seq analysis revealed a notable m^6^A modification site at the LINC00958 location. **B** RT-PCR indicated upregulated METTL3 mRNA expression in the BC cell line (MCF-7). **C** METTL3 knockdown (sh-METTL3) was transfected into MCF-7 cells to silence METTL3 expression. **D** M^6^A analysis (colorimetric method) presented m^6^A modification enrichment in sh-METTL3 or sh-NC transfection. **E** MeRIP-qPCR illustrated the m^6^A-modified LINC00958 level after METTL3 silencing or control. **F** RNA stability analysis demonstrated the LINC00958 level in MCF-7 cells transfected with METTL3 silencing. **p < 0.01 was considered as significant.

In the MeRIP-Seq analysis, our team found a notable m^6^A modification site at the LINC00958 location (Fig. 2A). Considering the existing report that the m^6^A methyltransferase METTL3 might install m^6^A on lncRNAs, we hypothesized that METTL3 could promote the m^6^A modification in LINC00958. In the BC cell line (MCF-7), the expression of the major m^6^A methyltransferase METTL3 was upregulated (Fig. 2B). Using METTL3 knockdown (Fig. 2C) transfection, m^6^A quantitative analysis showed that METTL3 silencing reduced m^6^A modification enrichment (Fig. 2D). MeRIP-qPCR illustrated that the m^6^A-modified LINC00958 level was decreased after METTL3 silencing (Fig. 2E). RNA stability analysis demonstrated that METTL3 silencing impaired the LINC00958 level, illustrating the role of METTL3 in LINC00958 overexpression in BC cells (Fig. 2F). Overall, these data suggested that m^6^A induced the upregulation of LINC00958 in BC cells.

### LINC00958 promoted the tumor progression of BC cells

**Fig. 3:**
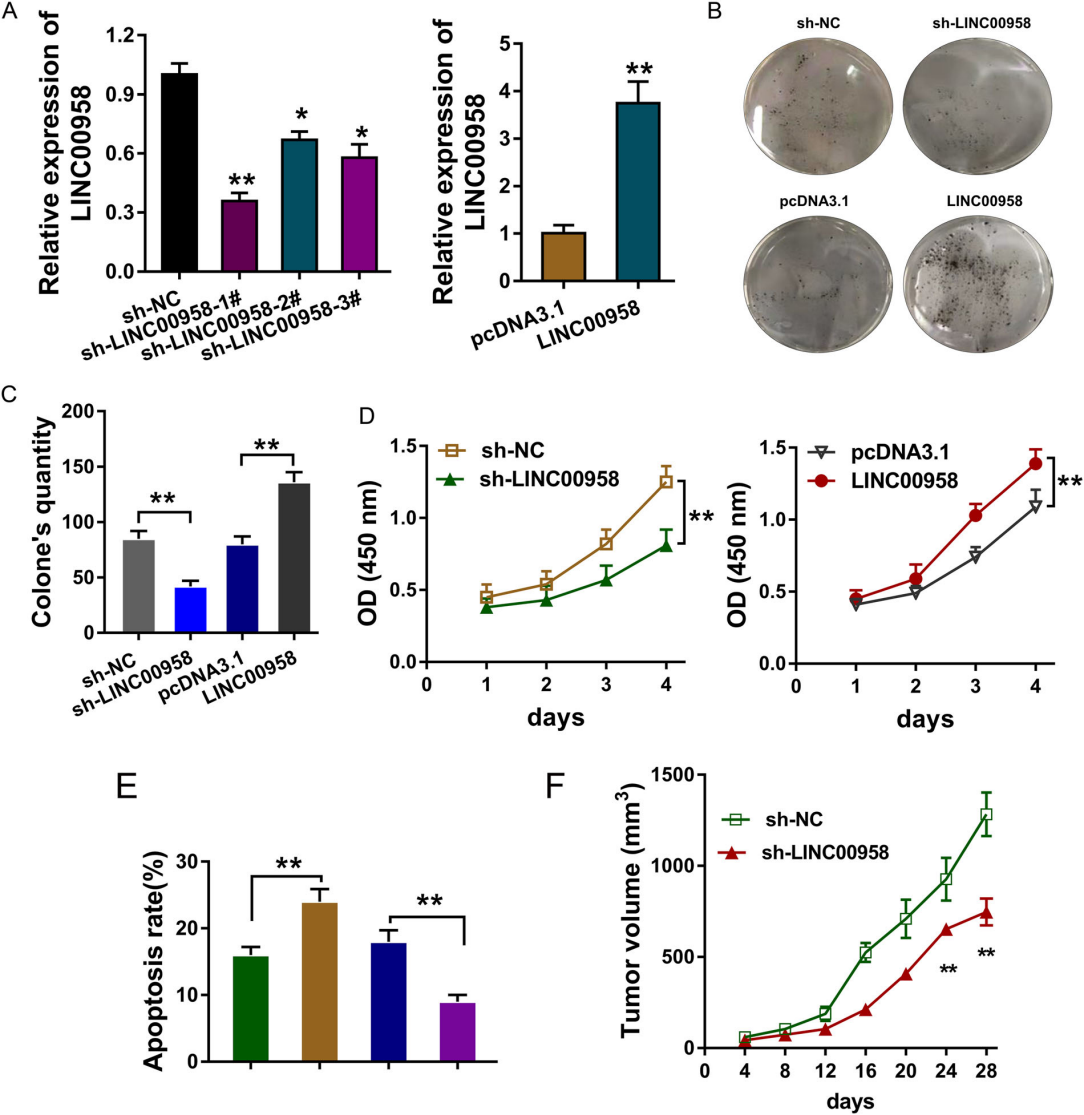
LINC00958 promoted the tumor progression of BC cells. **A** Gain- or loss-of-function assays were performed in MCF-7 cells transfected with sh-LINC00958 or LINC00958 overexpression vector. **B**, **C** Colony formation assay and **D** CCK-8 assays illustrated the proliferation of MCF-7 cells transfected with sh-LINC00958 or LINC00958 overexpression, as well as control groups. **E** Flow cytometry analysis of apoptosis demonstrated the apoptosis of MCF-7 cells transfected with sh-LINC00958 or LINC00958 overexpression. **F** In vivo mouse assays showed the inhibition of tumor growth by sh-LINC00958 transfection. **p < 0.01 and *p < 0.05 were considered significant.

The roles of LINC00958 in the BC cell phenotype were detected using gain- or loss-of-function assays in MCF-7 cells (Fig. 3A). Colony formation assays (Fig. 3B, C) and CCK-8 assays (Fig. 3D) illustrated that LINC00958 knockdown repressed the proliferation of MCF-7 cells, and the overexpression of LINC00958 promoted proliferation. Flow cytometry analysis of apoptosis found that LINC00958 knockdown accelerated the apoptosis of MCF-7 cells, and LINC00958 overexpression reduced apoptosis (Fig. 3E). In vivo mice injected with MCF-7 cells transfected with LINC00958 knockdown showed a remarkable inhibition of tumor growth (Fig. 3F). Taken together, these results indicated that LINC00958 promoted the tumor progression of BC cells.

### LINC00958 targeted miR-378a-3p

**Fig. 4:**
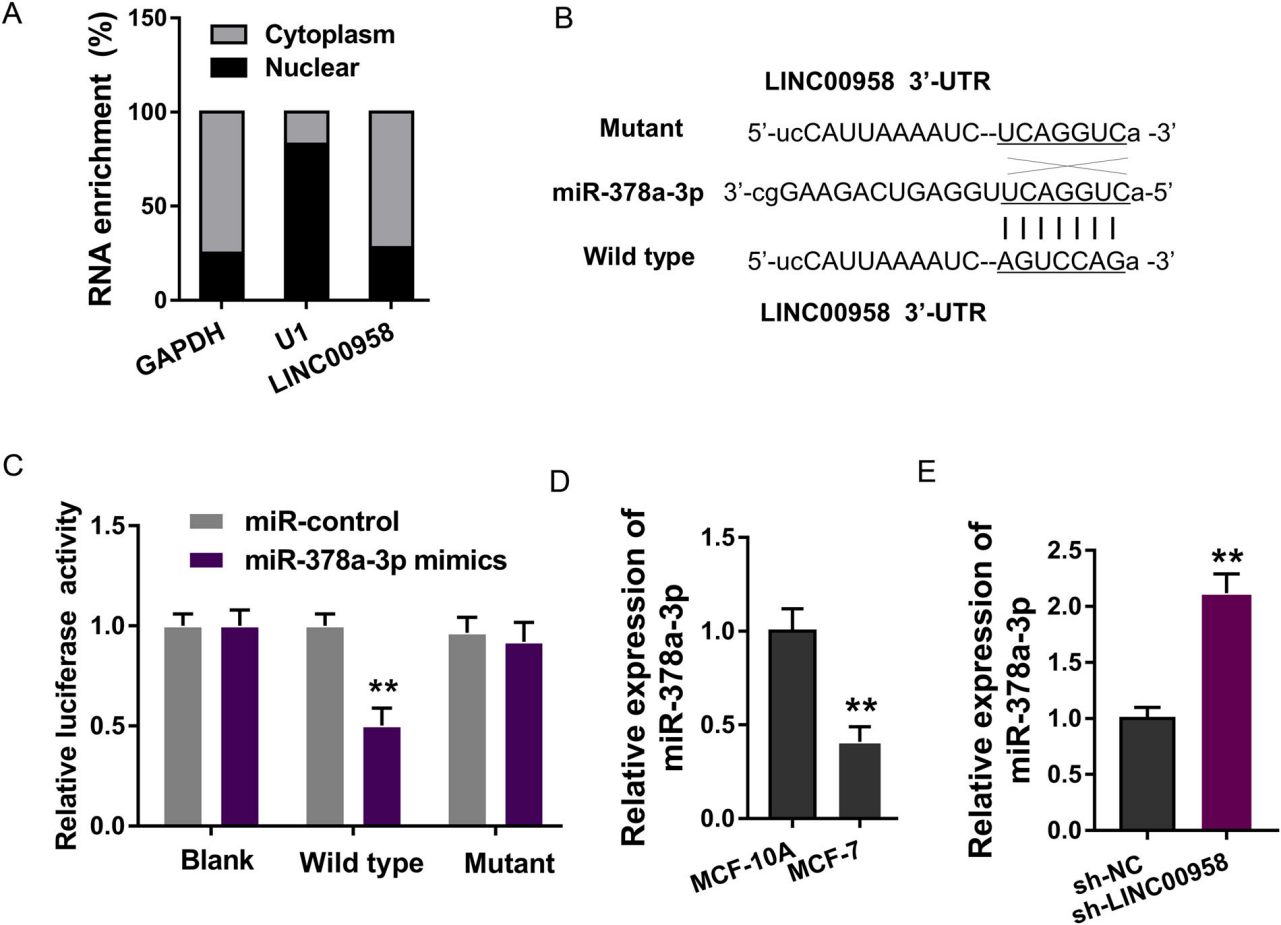
LINC00958 targeted miR-378a-3p. **A** Subcellular analysis found that LINC00958 was predominantly located in the cytoplasm of MCF-7 cells. **B** Bioinformatics predictive tools found targeting miR-378a-3p and LINC00958. **C** Luciferase reporter assay found that miR-378a-3p mimics could directly combine with wild-type LINC00958. **D** RT-PCR showed miR-378a-3p expression in BC cells (MCF-7) compared to normal cells (MCF-10A). **E** RT-PCR showed miR-378a-3p expression in MCF-7 cells transfected with LINC00958 knockdown. **p < 0.01 was considered as significant.

In the functional investigation of LINC00958, we found that LINC00958 was mainly distributed in the cytoplasm rather than the nucleus using subcellular fraction analysis (Fig. 4A). With the aid of bioinformatics predictive tools (StarBase, http://starbase.sysu.edu.cn/), we found that several miRNAs were potentially regulated by LINC00958. Among these miRNAs, miR-378a-3p demonstrated a notable connection with LINC00958 (Fig. 4B). A luciferase reporter assay found that miR-378a-3p mimics could directly combine with LINC00958 (Fig. 4C). RT-PCR showed that miR-378a-3p expression was decreased in BC cells (MCF-7) compared to normal cells (MCF-10A) (Fig. 4D). In MCF-7 cells, miR-378a-3p expression was upregulated in the LINC00958 knockdown transfection (Fig. 4E). In conclusion, these data supported that LINC00958 targeted miR-378a-3p in BC cells.

### LINC00958/miR-378a-3p targeted YY1

**Fig. 5:**
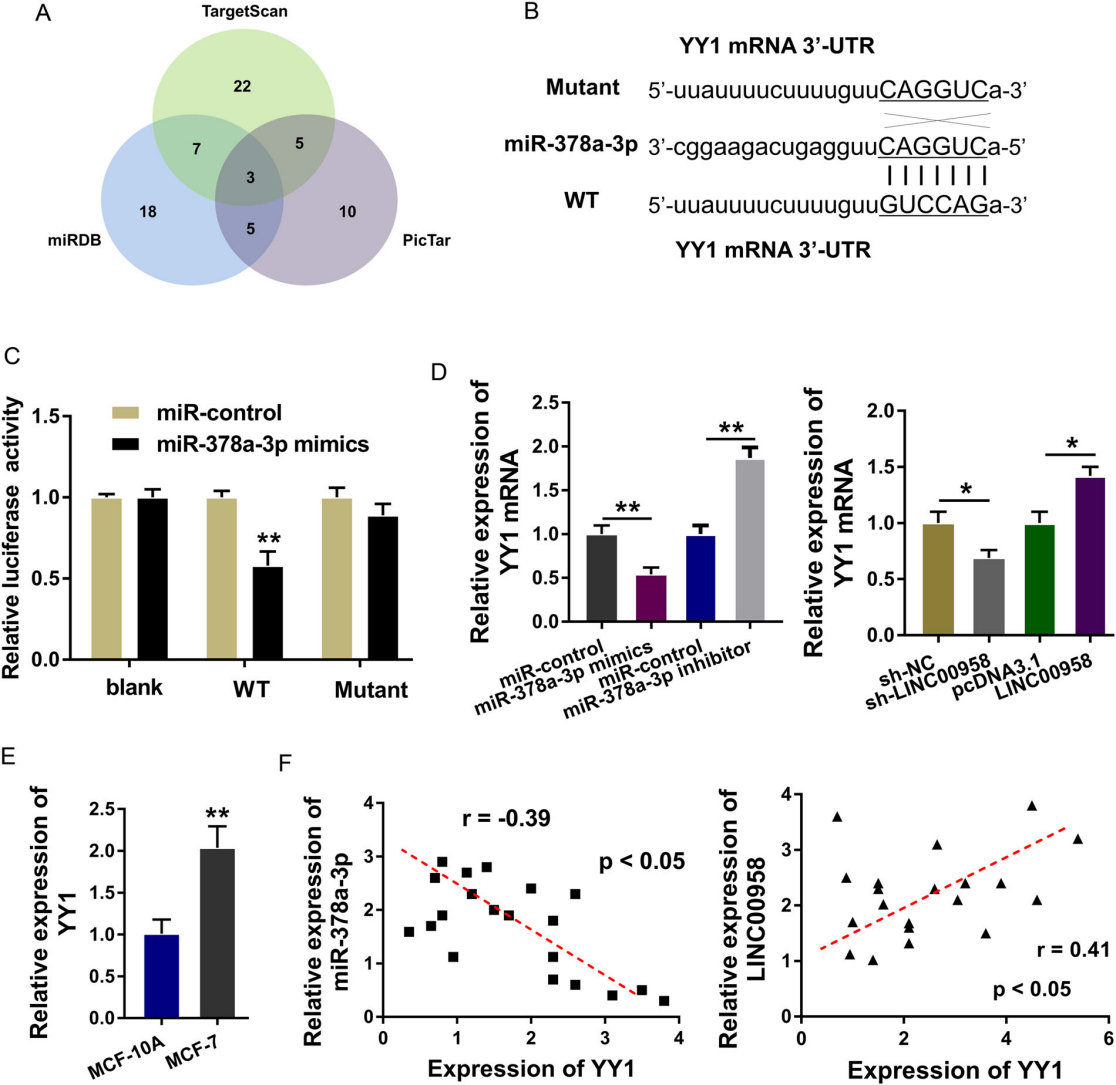
LINC00958/miR-378a-3p targeted YY1. **A** Bioinformatics predictive tools (TargetScan, miRDB, PicTar) identified multiple proteins that could be targeted by LINC00958/miR-378a-3p. **B** Luciferase reporter vectors for YY1 mRNA (wild type and mutant) were synthesized. **C** Luciferase gene reporter assay found that miR-378a-3p mimics/control connected with the YY1 mRNA wild type/mutant. **D** RT-PCR analysis found YY1 mRNA in MCF-7 cells transfected with miR-378a-3p mimics/inhibitor or LINC00958 knockdown/overexpression. **E** T-PCR analysis showed YY1 mRNA expression in MCF-7 cells and normal cells (MCF-10A). **F** Correlation analysis using Pearson’s *χ*^2^ method indicated the correlation with YY1 and LINC00958/miR-378a-3p. **p < 0.01 and *p < 0.05 were considered significant.

Bioinformatics predictive tools (StarBase, http://starbase.sysu.edu.cn/) found that there were multiple proteins that could be targeted by LINC00958/miR-378a-3p (Fig. 5A). Moreover, YY1 was found to be the target of LINC00958/miR-378a-3p, and luciferase reporter vectors (wild type and mutant) were constructed (Fig. 5B). A luciferase reporter assay found that miR-378a-3p mimics were tightly connected with wild-type YY1 mRNA (Fig. 5C). RT-PCR analysis found that miR-378a-3p mimic transfection decreased the YY1 mRNA expression and that the miR-378a-3p inhibitor upregulated the YY1 mRNA expression. Moreover, LINC00958 knockdown reduced the YY1 mRNA expression, and LINC00958 overexpression enhanced the YY1 mRNA expression (Fig. 5D). In MCF-7 cells, YY1 mRNA expression was markedly upregulated (Fig. 5E). Correlation analysis using Pearson’s *χ*^2^ method indicated that in BC samples, miR-378a-3p was negatively correlated with YY1 and LINC00958 was positively correlated with YY1 (Fig. 5F). In conclusion, these data supported that LINC00958/miR-378a-3p targeted YY1.

## Discussion

Given the critical role of epigenetic regulation in BC, lncRNAs play comprehensive roles in BC recurrence and metastasis^17,18^. Moreover, m^6^A acts as one of the most common RNA modifications in eukaryotes. Therefore, we focused on lncRNAs and their m^6^A modification, which might provide novel insight into BC. Hence, it is critical to reveal the underlying roles and mechanisms by which m^6^A-modified lncRNAs regulate BC tumorigenesis.

In the present study, we demonstrated that LINC00958 was markedly overexpressed in human BC tissues and indicated unfavorable BC prognosis. m^6^A RNA immunoprecipitation quantitative polymerase chain reaction (MeRIP-qPCR) showed that there was a notable m^6^A modification in LINC00958. Moreover, we found that the m^6^A methyltransferase METTL3 could install the methylation of LINC00958 and increase its expression. Thus, we summarized that m^6^A methylation induced the high expression of LINC00958 in BC. In addition, ectopic high expression of LINC00958 activated by m^6^A was also found in hepatocellular carcinoma. In hepatocellular carcinoma, METTL3-mediated m^6^A modification results in the upregulation of LINC00958 by stabilizing its mRNA, and LINC00958 sponges miR-3619-5p to upregulate hepatocellular carcinoma-derived growth factor (HDGF) expression to facilitate hepatocellular carcinoma progression and lipogenesis^19^.

Methyltransferase-like 3 (METTL3) acts as a critical catalyzing enzyme that is responsible for m6A modification^20–22^. In human cancer, an increasing number of reports have found that METTL3 can participate in the tumorigenesis process. For instance, Cai X et al.^3^ discovered that METTL3 promotes HBXIP expression through m^6^A modification, forming a positive feedback loop of HBXIP/let-7g/METTL3/HBXIP in BC^3^. For another example, Wang H et al.^14^ reported that METTL3 is overexpressed in BC and that the knockdown of METTL3 decreases proliferation and accelerates apoptosis by targeting Bcl-2^3^. Therefore, this evidence suggests that METTL3 participates in BC tumorigenesis.

In addition, we found that LINC00958 was distributed in the cytoplasmic portion and might function as a miRNA ‘sponge’. LINC00958 targeted miR-378a-3p to absorb its enrichment. Moreover, miR-378a-3p targeted the 3′-UTR of YY1 mRNA. Overall, we discovered the LINC00958/miR-378a-3p/YY1 axis in BC, which regulates proliferation and apoptosis, thereby promoting BC tumorigenesis progression. To date, a series of lncRNAs have been found to regulate BC tumorigenesis. For example, lncRNA LINC02273 is significantly elevated in metastatic lesions of BC tissue, and increased LINC02273 expression promotes BC metastasis through the recruitment of the hnRNPL-LINC02273 complex to the AGR2 promoter region^23^. LncRNA SNHG7, which is induced by c-Myc, is upregulated in both BC tissues and cell lines and targets the miR-34a-5p/lactate dehydrogenase A (LDHA) axis to promote glycolysis in BC^24^.

In conclusion, these research findings illustrated that LINC00958 was significantly upregulated in BC pathogenesis, and its upregulation promoted the tumorigenesis of BC cells. Mechanistically, METTL3-mediated m^6^A modification promotes the upregulation of LINC00958 by promoting its RNA transcript stability. Moreover, LINC00958 acted as a competitive endogenous RNA for miR-378a-3p to positively regulate YY1. Taken together, these findings provide novel insight into how m^6^A-mediated increases in LINC00958 expression regulate BC tumorigenesis and provide a potential therapeutic target for BC.

## Materials and methods

### Human tissue

A total of thirty patients with BC who underwent surgical resection were recruited for this study. Tumor tissues were obtained during the surgery and stored at −80 °C for further assays. None of these volunteers received chemotherapy or radiotherapy before this study. Paired adjacent noncancerous tissues served as the controls. The study was approved by the Ethics Committee of the First Hospital of Shanxi Medical University. All recruited patients were informed of this clinical study and signed informed consent before surgery. The clinicopathological characteristics are displayed in Table 1.

**Table 1:** Clinicopathological feature of BC patients with high/low expression of LINC00958.

	Total	LINC00958		*p*
		Low = 13	High = 17	
Age (years)				0.603
≥50	17	7	10	
<50	13	6	7	
TNM				0.016^*^
I-II	15	4	11	
III/IV	15	9	6	
Lymph metastasis				0.423
No	13	6	7	
Yes	17	7	10	
Tumor size				0.368
<2 cm	14	7	7	
≥2 cm	16	6	10	
Differentiation				0.298
well, moderate	17	7	10	
poor	13	6	7	

^*^P < 0.05 represents statistical difference. High or low LINC00958 expression was divided according to the median value. The optimal cutoff value of the relative expression of LINC00958 in BC was identified using ROC curve analysis in Cutoff Finder (http://molpath.charite.de/cutoff/).

### Cell and culture

Normal mammary cells (MCF-10A) and BC cell lines (MDA-MB-231, BT-549, MCF-7, and MDA-MB-468) were purchased from the Chinese Academy of Sciences Cell Bank and Cellbio (China). The cells were cultured according to the suppliers’ instructions. The cells were cultured in DMEM supplemented with 100 U/ml penicillin, 300 μg/l L-glutamine, and 10% FBS in a 5% CO_2_ incubator at 37 °C.

### RNA oligoribonucleotides and transfection

RNA oligoribonucleotides targeting LINC00958 (sh-LINC00958 and controls) and miR-378a-3p (mimics and control) were generated using synthetic short hairpin RNA oligo (RiboBio Co., Guangzhou, China). The LINC00958 complementary DNA (pcDNA-LINC00958) fragment and control vectors were also provided by RiboBio. MCF-7 cells (1 × 10^5^/well) were plated in 24-well plates, and 100 nM RNA oligoribonucleotides were transfected using Lipofectamine 2000 (Invitrogen) according to the manufacturer’s instructions. For METTL3 knockdown, shRNAs targeting METTL3 and controls were constructed by annealing the double-strand hairpin vector, and transfection was performed according to the manufacturer’s protocol.

### RNA isolation and quantitative real-time PCR

TRIzol reagent (Invitrogen) was first used to isolate total RNA according to the manufacturer’s protocol. The SuperScript First-Stand Synthesis system (Invitrogen, US) was used to prepare first-strand complementary DNA (cDNA). FastStart Universal SYBR Green Master Mix (Roche) was used to prepare real-time PCR on an ABI 7500 Fast Real-Time PCR system (Applied Biosystems, USA). For the quantification of lncRNA, miRNA and mRNA expression, relative data were calculated by the 2^-ΔΔCt^ statistic method normalized to GAPDH or U6. All these primers were provided by GenePharma (Shanghai, China) and are available in Table S1 supplementary materials.

### Cell proliferation

Cell proliferation was detected using CCK-8 and colony formation assays. First, MCF-7 cell growth (2000 cells/well) was quantified using Cell Counting Kit-8 (CCK-8, Beyotime Corporation, Shanghai, China). Second, in the colony formation assay, the cells were replaced with medium and cultured at 37 °C for 14 days. Then, cell clones were fixed with methanol for 10 min and stained with crystal violet for 20 min. Cells were counted under an inverted microscope.

### Flow cytometry

Cell apoptosis was analyzed by flow cytometry according to the manufacturer’s recommendation. In brief, transfected cells were harvested and administered propidium iodide and fluorescein isothiocyanate (FITC)-annexin V using the FITC Annexin V Apoptosis Detection Kit (BD Biosciences). Early apoptotic cells and late apoptotic cells were calculated using FACS Canto II flow cytometry (BD Biosciences).

### m^6^A quantitative analysis

Total RNA was isolated using TRIzol (Invitrogen) according to the manufacturer’s instructions, and its quality was analyzed by NanoDrop. The m^6^A quality in the total RNAs was detected using an m^6^A RNA methylation quantification kit (ab185912; Abcam). Finally, the m^6^A content in the total RNA was quantified colorimetrically by measuring the absorbance at 450 nm.

### m^6^A RNA immunoprecipitation quantitative polymerase chain reaction (MeRIP-qPCR)

For quantification of m^6^A-modified LINC00958 levels, MeRIP-qPCR was performed as previously described^25^. First, total RNA was isolated from BC cells by TRIzol. Anti-m^6^A antibody (Abcam, ab208577) or anti-IgG interacted with protein A/G magnetic beads in IP buffer overnight. Then, the RNA was incubated with antibody-coated magnetic beads in IP buffer. After precipitation and elution, the m^6^A level was detected as the relative value.

### RNA stability assay

To detect RNA stability, MCF-7 cells were treated with actinomycin D (1 μg/ml), and RNA was extracted at different time points for qRT-PCR.

### Subcellular location analysis

The separation of the nuclear and cytosolic LINC00958 fractions was carried out using the PARIS Kit (Life Technologies). The separated RNA was detected using real-time PCR.

### Dual-luciferase reporter assay

A dual-luciferase reporter assay was performed to identify the direct interaction within LINC00958, miR-378a-3p, and YY1. The 3′-untranslated region of LINC00958 with a potential binding site targeting miRNA was cloned into the psiCheck2 (Promega) dual-luciferase reporter gene, including wild-type (WT) and mutant (Mut) LINC00958. 293 T cells were transfected with miRNA mimics and negative controls with luciferase vectors. The Renilla luciferase assay system (Promega, Madison, WI) was used to quantify Renilla luciferase activity.

### Xenograft mouse in vivo model

The protocol was approved by the Committee on the Ethics of Animal Experiments of First Hospital of Shanxi Medical University. Five-week-old BALB/c nude male mice (18–22 g) were provided by Vitalstar Biotechnology Co., Ltd. (Beijing, China) and maintained in pathogen-free conditions. MCF-7 cells transfected with sh-LINC00958 or empty vector were resuspended at 2 × 10^7^ cells/mL. Mice were subsequently injected in the flank. Tumor growth was detected every three days for tumor volumes using Vernier calipers. The formula was length × width^2^ × 0.5. This animal study was performed in strict accordance with the Guide for the Care and Use of Laboratory Animals of the NIH.

### Statistical analysis

The chi-square test was used to investigate the association between LINC00958 and clinicopathological characteristics. In addition, a *t*-test was performed to compare the mRNA or ncRNA expression levels in different groups. Kaplan–Meier curves and log-rank tests were performed to compare the BC survival outcomes. Statistical analysis was performed by using SPSS 18.0 statistical software (SPSS, Chicago, IL, USA), with a *p*-value less than 0.05 considered statistically significant.

## Supplementary information

Figure S1

Figure S2

Figure S3

Figure S4

Table S1

## Data Availability

The authors have no research data to share.
