# Supplementary figures and images for: m6A-induced LINC00958 promotes breast cancer tumorigenesis via the miR-378a-3p/YY1 axis
Source: Cell Death Discov. 2021 Feb 2;7:27. doi: 10.1038/s41420-020-00382-z (PMC7854648; doi:10.1038/s41420-020-00382-z)

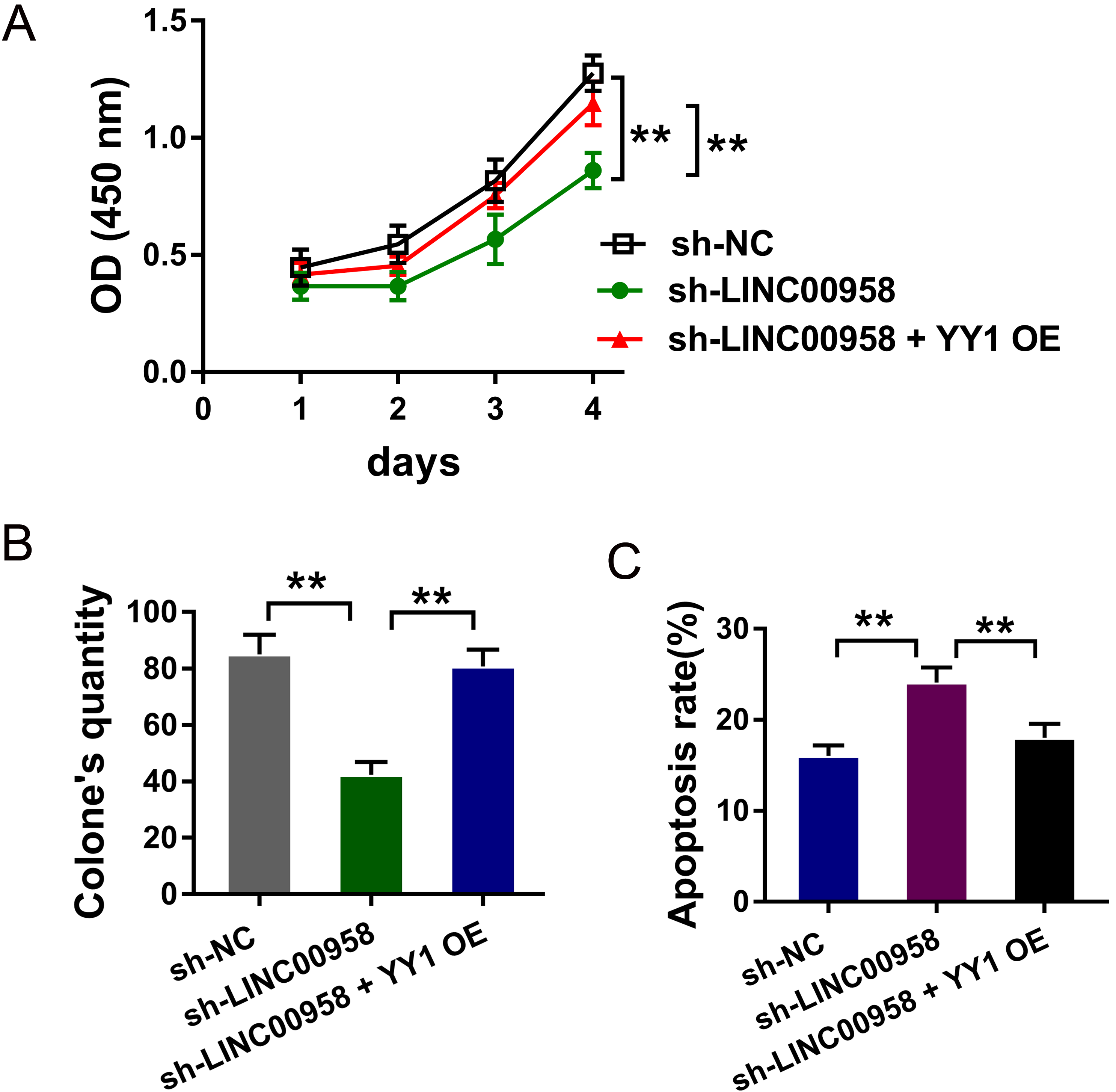

Supplement: Supplementary file 1 — Figure S1 [file 41420_2020_382_MOESM1_ESM.tif]

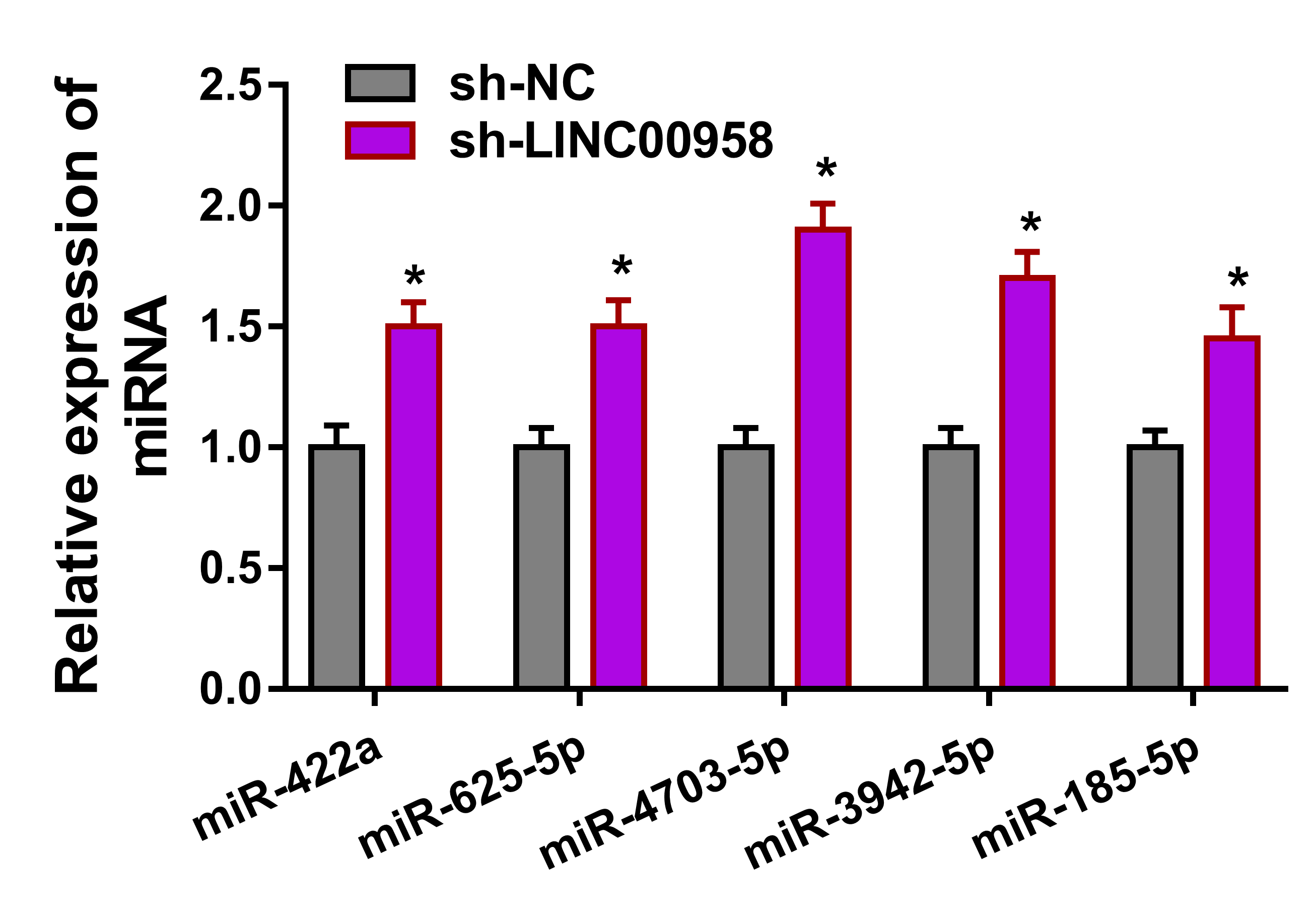

Supplement: Supplementary file 2 — Figure S2 [file 41420_2020_382_MOESM2_ESM.tif]

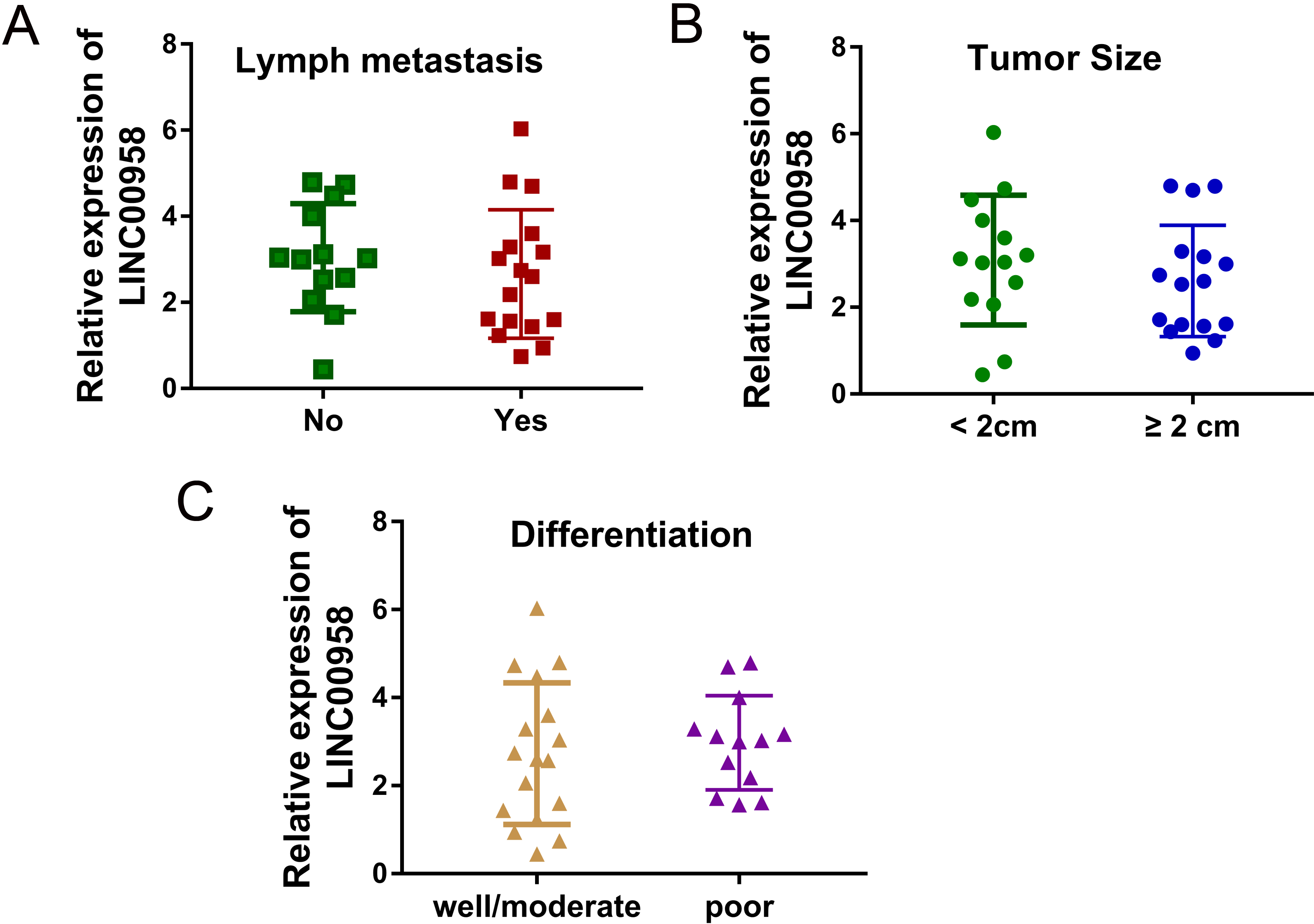

Supplement: Supplementary file 3 — Figure S3 [file 41420_2020_382_MOESM3_ESM.tif]

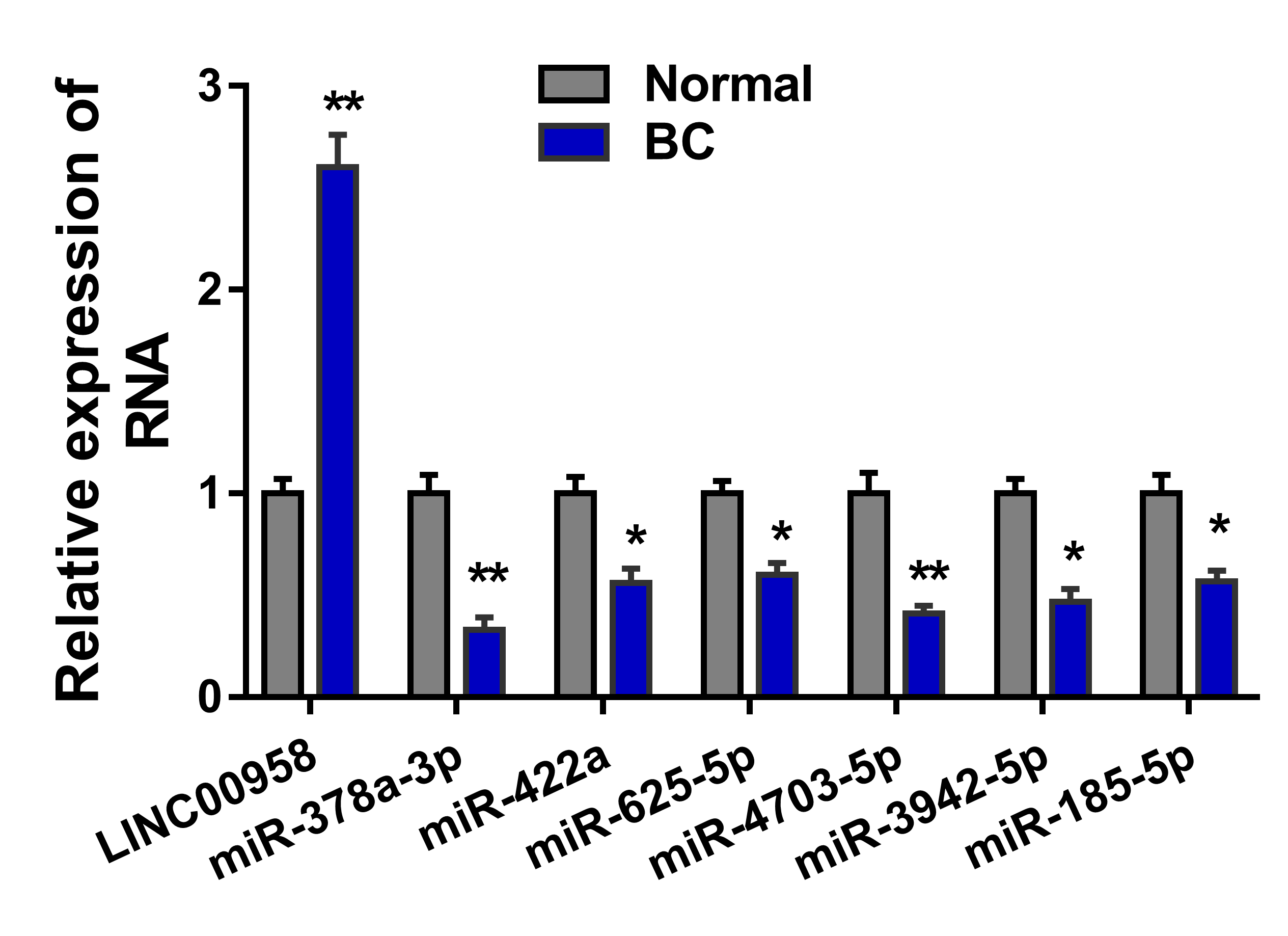

Supplement: Supplementary file 4 — Figure S4 [file 41420_2020_382_MOESM4_ESM.tif]
