# Supplementary material for: m6A-induced LINC00958 promotes breast cancer tumorigenesis via the miR-378a-3p/YY1 axis
Source: Cell Death Discov. 2021 Feb 2;7:27. doi: 10.1038/s41420-020-00382-z (PMC7854648; doi:10.1038/s41420-020-00382-z)
Supplement: Supplementary file 5 — Table S1 [file 41420_2020_382_MOESM5_ESM.docx]

**supplemental Table S1**. Sequences of shRNA and qRT-PCR primers.

|  | 5’-3’ |
| --- | --- |
| LINC00958 | forward, 5’-GCCTGGCACATTCAGTGGAGAG-3’  reverse, 5'-GTGGCGGCCTGAGCTTCTTC-3’ |
| miR-378a-3p | 5’-CCCCTATCACGATTAGCATTAA-3’ |
| YY1 | forward, 5’-ACGGCTTCGAGGATCAGATTC-3’  reverse, 5’-TGACCAGCGTTTGTTCAATGT-3’ |
| METTL3 | forward, 5’-CTATCTCCTGGCACTCGCAAGA-3’  reverse, 5’-GCTTGAACCGTGCAACCACATC-3’ |
| beta-actin | forward, 5’-CTCCATCCTGGCCTCGCTGT-3’  reverse, 5’-GCTGTCACCTTCACCGTTCC-3’ |
| sh-LINC00958-1 | 5’-GAGGTACCCAATAGTTTCATT-3’ |
| sh-LINC00958-2 | 5’-ATGCCAAGTTATTCCAACTTT-3’ |
| sh-LINC00958-3 | 5’-GTACCCAAGTTATTCAGGATT-3’ |
| miR-378a-3p mimics | 5’-AUCCGACGGUCCGAAUAAG-3’ |
| miR-378a-3p inhibitor | 5’-UGGAGAUUGGACCGGAACC-3’ |
| sh-METTL3 | CCGGGCCTTAACATTGCCCACTGATCTCGAGATCAGTGGGCAATGTTAAGGCTTTTTG |
